# The Exploration of Disease Pattern, Zheng, for Differentiation of Allergic Rhinitis in Traditional Chinese Medicine Practice

**DOI:** 10.1155/2012/521780

**Published:** 2012-07-29

**Authors:** Sienhung Yang, Hsingyu Chen, Yihsuan Lin, Yuchun Chen

**Affiliations:** ^1^Division of Chinese Internal Medicine, Center for Traditional Chinese Medicine, Chang Gung Memorial Hospital, No. 123, Dinghu Road, Guei-shan, Taoyuan 33378, Taiwan; ^2^School of Traditional Chinese Medicine, College of Medicine, Chang Gung University, No. 259, Wen-Hwa 1st Road, Guei-shan, Taoyuan 333, Taiwan; ^3^Graduate Institute of Clinical Medical Sciences, College of Medicine, Chang Gung University, Taoyuan, Taiwan; ^4^Department of Medical Research and Education, National Yang-Ming University Hospital, I-Lan, Taiwan

## Abstract

Pattern, or “zheng,” differentiation is the essential guide to treatment with traditional Chinese medicine (TCM). However, the considerable variability between TCM patterns complicates evaluations of TCM treatment effectiveness. The aim of this study was to explore and characterize the relationship between patterns and the core patterns of allergic rhinitis. We summarized 23 clinical trials of allergic rhinitis with mention of pattern differentiation; association rule mining was used to analyze TCM patterns of allergic rhinitis. A total of 205 allergic rhinitis patients seen at Chang Gung Memorial Hospital from March to June 2005 were included for comparison. Among the 23 clinical trials evaluated, lung qi deficiency and spleen qi deficiencies were the core patterns of allergic rhinitis, accounting for 29.50% and 28.98% of all patterns, respectively. A higher prevalence of lung or spleen qi deficiency (93.7%) was found in Taiwan. Additionally, patients with lung or spleen qi deficiency were younger (27.99 ± 12.94 versus 58.54 ± 12.96 years) and the severity of nasal stuffiness was higher than among patients with kidney qi deficiency (1.35 ± 0.89 versus 0.62 ± 0.65; *P* < 0.05). Lung and spleen qi deficiencies are the core patterns of allergic rhinitis and determining the severity of nasal stuffiness is helpful in differentiating the TCM patterns.

## 1. Introduction

Traditional Chinese medicine (TCM) has been used for centuries in China and more recently has been widely studied and applied throughout the world [1, 2]. “Pattern differentiation and treatment” has an important role in TCM treatment. With this approach, a diagnosis is established through four examinations: visual inspection, smelling and listening, inquiry, and palpation, followed by TCM interventions such as use of herbal medicine, acupuncture, moxibustion, and massage [3, 4].

Pattern differentiation, or “zheng,” is a unique TCM concept that summarizes the nature, location, and pattern of diseases corresponding to the World Health Organization's definition [4]. According to each individual pattern, the specific TCM treatment can be prescribed precisely to maximize its effectiveness [5–7]. However, successful use of pattern differentiation depends primarily on TCM doctors' subjective judgment, which is based upon classical TCM principles, education, and clinical experience. Thus, the practice of pattern differentiation can vary considerably among individual physicians [8]. In addition, there is little agreement between textbook guidelines for TCM pattern differentiation and its actual use in practice [9]. Finding ways to incorporate TCM knowledge into clinical practice and eliminating variability is an important issue in evidence-based investigations [9].

Due to the considerable variability in individual practices, it can be difficult to summarize TCM clinical data by conventional statistical techniques, and thus a number of data mining methods, such as association rule mining (ARM) and cluster analysis, are used to acquire TCM knowledge from large-scale clinical data [3, 10, 11]. ARM is a modern data mining tool developed to explore the relationships between a wide range of factors, and it is widely applied to TCM prescription analysis [10, 12]. Moreover, ARM can effectively pinpoint the core TCM formula from a large prescription database by analyzing the relationship between TCM formulas [11]. In addition to TCM prescription, ARM is also used to analyze disease comorbidities and TCM patterns, and the advantages in reducing the complexity of TCM patterns have been well demonstrated [13, 14].

Allergic rhinitis, a common immunologic disorder, affects 10% to 20% of the world's population [15]. It involves type 2 CD4 T lymphocyte activation with cytokine secretion, producing an increased number of eosinophils and mast cells. Certain drugs used in Western medicine (WM), such as H_1_-antihistamines, leukotriene receptor antagonists, intranasal corticosteroids, and even short-term oral corticosteroids, have been used to block disease progression and relieve symptoms [15]. In Taiwan, allergic rhinitis is one of the most common reasons for TCM visits, due to concern about side effects from long-term use of Western medications and the prospect of fewer side effects with TCM treatment [2, 16].

Several TCM treatments have been beneficial for allergic rhinitis, and the results of many studies have outlined the possible mechanisms for suppressing allergic reactions [17–21]. Nonetheless, the effectiveness of different TCM treatments is still unclear because no large-scale survey on TCM pattern differentiation of allergic rhinitis has yet been done. 

The aim of this study was to explore the core TCM patterns of allergic rhinitis by using ARM and to compare these results with a hospital-based database to identify crucial factors to differentiate the patterns of allergic rhinitis. Depending upon the results of this study, future studies could focus on the most important TCM patterns, and different treatments could then be designated for specific TCM patterns.

## 2. Materials and Methods

### 2.1. Construction of the Clinical Trial Database

First, we conducted an extensive search of several databases, including PubMed, MEDLINE, Web of Science, Scopus, and the China Academic Journals Full-Text Database (CJFD). Keywords searched included “allergic rhinitis,” “bi qiu,” “chronic rhinitis,” “pattern differentiation,” “syndrome differentiation,” “zheng,” and “clinical trials.” “Bi qiu” is the TCM disease corresponding to allergic rhinitis in WM. The full text of the search results was accumulated and critiqued by all authors of this study, and disagreements were resolved by consensus. After critical appraisal, the essential elements, including case number, gender, age, diagnostic criteria, and distribution of TCM patterns, were extracted from the eligible clinical trials manually. All these elements were entered into the computerized database.

### 2.2. Association Rule Mining (ARM)

ARM, a data mining technique developed in the 1990s, has been widely used in medical research to explore the relationships among TCM prescriptions, disease comorbidities, and TCM patterns [13, 14]. The detailed algorithm has been thoroughly described and presented in previous studies, and IBM DB2 Intelligent Miner 9.1 software (IBM Corporation, Armonk, NY) was used to perform ARM of the clinical trials database [22]. Two decisive factors, support and confidence, were used to demonstrate relationships between patterns. Support was defined as the prevalence of a certain relationship among the whole database, and conditional probability of coexistence of pattern A and B given only pattern A was related to confidence. Depending on the threshold formed both by the support and confidence factors, the significant relationship between pattern A and B was established. It was an iterative process to decide the proper value of support and confidence factors and, in this study, support and confidence factors were set to 1% and 20%, respectively. These values were agreed upon by all authors in this study. Additionally, a diagram was drawn of associations between all patterns to clarify the relationships between TCM patterns and the core patterns of allergic rhinitis. 

### 2.3. Hospital-Based Clinical Data Acquisition

To compare ARM results from the clinical trials database and practical clinical data, we used an established database of allergic rhinitis patients in the TCM outpatient service at CGMH. The definitive diagnosis of allergic rhinitis and TCM patterns was confirmed by Dr. Yang. Detailed data, including TCM patterns, age, gender, parents' health history, patients' personal health history, residence, serum IgE levels, results of MAST (Multiple Allergen Simultaneous Test panel) tests, and symptom severity, were recorded in this database. All data were collected with informed consent, and the records from March to June 2005 were extracted for further analysis. The process of data collection and analysis was approved by the Institutional Review Board (IRB) of CGMH.

### 2.4. Statistical Analysis of Characteristics of TCM Patterns

To examine the differences in characteristics among TCM patterns Student's *t*-test and one-way analysis of variance (ANOVA) were used for numerical data, and chi-square statistics were applied to categorical data. Only results of statistics with a *P *value less than 0.05 were deemed to be significant. 

## 3. Results

### 3.1. Description of Clinical Trials of TCM Patterns

A total of 114 studies were found by the search strategy, and after detailed appraisal, 23 studies were eligible for inclusion in the study. All 23 studies were done in China and had been published in Chinese. Studies with English titles are listed as examples in the Appendix. From the 23 eligible studies, 2589 patients were identified, and a patient-pooled database was constructed. Fifteen patterns composed of one or more organs and the nature of disease were identified. Lung qi deficiency was the most common pattern (23.95%), followed by spleen qi deficiency (22.75%), and lung yang deficiency with wind-cold assailing the lung (14.75%). More than half of patients were classified into the qi deficiency pattern in these trials. In contrast, blood stasis, dual deficiencies of qi and yin of lung, and lung-spleen yang deficiency were the least-recognized patterns, and all had a prevalence of less than 1% (Table 1).

### 3.2. ARM of TCM Patterns

**Figure 1:**
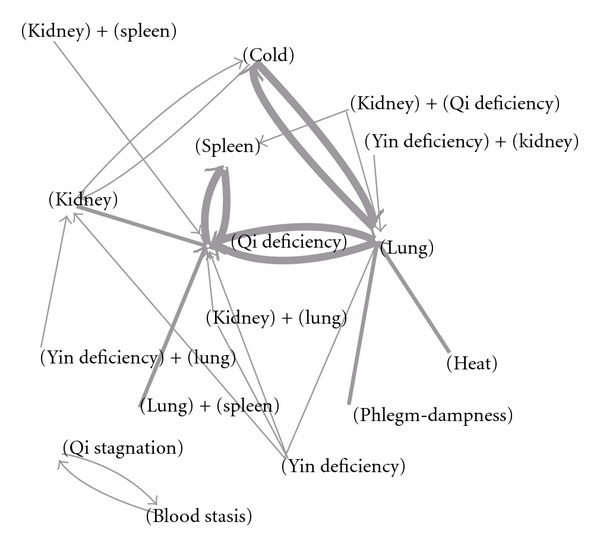
The associations between different TCM patterns of allergic rhinitis. Note: the width of connection lines represents the prevalence of the associations.

After applying ARM, we identified the 10 most common relationships between the locations and nature of disease patterns (Table 2). The lung, followed by the spleen, was the most common site of disease, whereas qi deficiency was the most common nature of disease. More than half (58.48%) of all pattern combinations were composed of lung or spleen qi deficiency. Nearly all locations or cases of allergic rhinitis were connected to the lung, spleen, and qi deficiency, and strong interactions were also found. The central role of the lung and spleen can be seen in a diagram of relationships between patterns (Figure 1).

Additionally, high confidence, as high conditional probability, was found among three conditions: “heat with lung,” “phlegm-dampness with lung,” and “kidney and spleen with qi deficiency.” It is assumed that, for patients with allergic rhinitis, once heat or phlegm-dampness was found, the nature of these two diseases would always be combined with lung, forming a pattern. More interestingly, qi stagnation and blood stasis were strongly associated, and neither had any relationship with major organs, such as lung, spleen, or even kidney. Despite the fact that this group's prevalence was only 0.19%, it may represent special mechanisms or manifestations of allergic rhinitis. 

### 3.3. Pattern Analysis in Hospital-Based Surveillance

Using the well-established allergic rhinitis patient database at CGMH, TCM pattern analysis showed these patients could be divided into 3 groups: those with lung qi deficiency, dual deficiency of lung-spleen qi, and kidney qi deficiency (Table 3). Similar to the results of clinical study reviews done in China, 93.7% of patients had patterns composed of lung, spleen, and qi deficiency, and the percentage was higher than in the clinical trials. Among all the patients' characteristics, patients diagnosed with kidney deficiencies were significantly older than the other two groups—57.37 years versus 27.99 years—whereas no differences were found in serum IgE levels, results of MAST allergy tests, or other factors (Table 3). 

### 3.4. Relations between TCM Patterns and Symptoms

TCM pattern differentiation was mainly based on clinical symptoms and therefore analysis of patients' symptom severity provided decisional information for pattern differentiation. Higher symptom severity scores, equivalent to more severe symptoms, were noted in the lung qi deficiency group and dual deficiency of the lung-spleen qi group, compared to the kidney qi deficiency group, although this was not statistically significant (Table 3). Nevertheless, the differences in symptom severity became more obvious when lung and spleen qi deficiency were combined due to symptom similarity, and compared with the kidney qi deficiency group (Table 4). Moreover, “stuffiness,” one of the most bothersome effects of allergic rhinitis, was found to be more severe in the lung or spleen qi deficiency group than in the kidney qi deficiency group (Table 4). 

## 4. Discussion

To the best of our knowledge, this is the first study to investigate the TCM patterns of clinical trials and to provide comparisons of clinical hospital-based data and severity of symptoms. The use of TCM has become much more widespread in recent years and many more interventions guided by TCM theory are being integrated into modern medicine [1, 2, 9]. TCM treatments, including herbal medicine, acupuncture, moxibustion, and massage, are administered according to TCM patterns, or “zheng” [23]. TCM patterns are composed of the cause, nature, and location of diseases, and differentiation of patterns is largely dependent upon clinical symptoms [3, 24]. Because of the complexity and plurality of clinical symptoms, and the nature and location of diseases, such as the Chinese medicine theory of five viscera and six bowels, the variability of pattern differentiation is extremely high. Thus, agreement on patterns of the same disease is usually low [8, 9].

From the viewpoint of evidence-based medicine, in future studies, it will be particularly important to summarize TCM patterns and to explore core patterns of disease. ARM is an appropriate statistical method for summarizing disease patterns and exploring core patterns and the nature and locations of diseases because it examines not only the prevalence of a pattern but also the strength of relations between and within patterns [14]. In this study, combinations of lung, spleen, and qi deficiencies were found to be the most crucial part of TCM patterns of allergic rhinitis. The results are consistent among clinical trials and hospital-based clinical data, and disclose valuable, evidence-based information for further investigation.

Qi deficiency has been proved to be crucial to allergic rhinitis in previous studies, and two famous qi-tonifying Chinese herbal products, Bu-zhong-yi-qi-tang and Xiang-sha-liu-jun-zi-tang, have had marked therapeutic effects on allergic rhinitis, even without pattern differentiation [18–20]. The mechanisms of immunomodulation of qi-tonifying agents include decreasing serum IgE, interleukin-4 (IL-4), interleukin-5 (IL-5), and gamma interferon (IFN-*γ*), increasing interleukin-10 (IL-10), and suppressing cyclooxygenase 2 mRNA expressions [18–20]. As a result, the imbalance of type 1 and type 2 helper T lymphocyte cells is reversed and allergic rhinitis symptoms are alleviated [18, 20]. IL-4 and IL5 with helper T-lymphocytes switch from type 1 to type 2, and subsequently high IgE secretion has been proved to be the cardinal pathogenesis of an allergic reaction [25–27]. The effective reversal of activation of an allergic reaction by qi-tonifying agents shows the possible relationship between qi deficiency and serum cytokine level, and, perhaps, the pathogenesis of qi deficiency of allergic rhinitis. 

Lung and spleen are the two important locations of diseases and are highly related to qi deficiency, forming TCM patterns. The function of lung, from the viewpoint of TCM, includes control of respiration, qi domination, and fluid regulation, and these functions are highly related to the nose and skin [4]. The most common symptoms of allergic rhinitis, such as sneezing, runny nose, and stuffiness, and possible subsequent critical illness in the form of asthma have been shown to be associated with the nose and entire respiratory tract and share the similar pathogenesis [15]. Moreover, immunomodulation of allergic diseases by lung-tonifying agents such as *Astragalus membranaceus *and *Cordyceps militaris* has been widely reported [28, 29]. Owing to the remarkably similar disease behavior and pathogenesis, the lung, rather than other organs, represents the most important organ in pattern differentiation of allergic rhinitis. 

The spleen, from the viewpoint of TCM, dominates transformation of food to energy, similar to WM's view of the gastrointestinal tract's function [4]. The gastrointestinal system has been thought to be associated with allergic diseases and the underlying mechanism may be related to activation of eosinophils and type 2 helper T lymphocytes, with increasing IgE levels [30, 31]. Thus, by modifying intestinal bacterial flora and subsequent systemic immunomodulation, symptoms of allergic rhinitis may be relieved [32]. Additionally, a spleen-tonifying TCM formula has been found to be effective for alleviating allergic rhinitis symptoms [33]. These facts reveal the close relationship between spleen deficiency and allergic reactions, and through modulating gastrointestinal function by TCM herbal products, allergic disorders may be alleviated. 

Yin and yang deficiencies are less commonly identified than qi deficiency in clinical trials, and they were also absent in the surveillance at our hospital. Yin deficiency was a specialized TCM pattern characterized by decreased body fluids, and it was diagnosed when patients complained about dryness of the mouth, throat, and nasal passages, or constipation. Additionally, a reddish tongue with scanty coating and a fine, rapid pulse were commonly seen among such patients. Moreover, symptoms of yang deficiency among allergic rhinitis patients included manifestations of qi deficiency with prominent fear of cold, cold extremities, clear nasal discharge, pale face, and an enlarged tongue with a white, slick coating. Both lung yin and yang deficiencies were noted in the late stage of the clinical course of allergic rhinitis, and they usually developed when qi deficiency, the early stage of allergic rhinitis, was not properly treated. Therefore, it is reasonable that combinations of qi and yin deficiency or yang deficiency were less frequently found among allergic rhinitis patients.

Additionally, combination of qi stagnation and blood stasis was a special pattern in this study. Although the prevalence was low, about 1.78%, a strong association with allergic rhinitis was found (Tables 1 and 2). Also, this group of patients seemed to be isolated from other patients (Figure 1). In other words, once qi stagnation was diagnosed, blood stasis was always also diagnosed, and vice versa. Qi stagnation and blood stasis among allergic rhinitis patients had a chronic course, and patients had a purplish or purple-spotted tongue and a stringy, choppy pulse. Due to the unusual characteristics, a different pathogenesis was suspected among these patients and therefore further studies were warranted.

The severity of nasal stuffiness, one of the common symptoms of allergic rhinitis, is definitely different between lung or spleen qi deficiency and kidney qi deficiency groups. In this study, the patients in the kidney qi deficiency group were older than those in the lung or spleen qi deficiency group. This finding is similar to that of previous studies. Currently, nasal stuffiness is thought to be caused by eosinophil and mast cell infiltration with subsequent airway remodeling. It is believed to be related to certain neuropeptides, and its severity decreases with aging [34]. From TCM's viewpoint, metabolism and transport of body fluids largely depend on lung and spleen [4] and therefore nasal stuffiness, caused by nasal cavity mucosa edema and swelling due to allergic reaction, is easily found in patients with lung and spleen qi deficiency with disturbed body fluid transport. Additionally, the prominent immunologic disorder found among lung and spleen qi deficiency patients may also be the cause of severe nasal stuffiness. Based on this significantly different symptom among the two groups, nasal stuffiness can be used as an inclusion or exclusion criteria for patient selection, and different treatment plans are able to be individually provided for the specific groups. 

Though the clinical data are closely comparable to the summarized results of clinical trials for allergic rhinitis, there are still some limitations to this study. First, the quality of clinical trials is heterogeneous. Some population characteristics, such as gender, age, or detailed manifestations of allergic rhinitis, are not provided in every trial, and therefore selection bias may exist. To effectively eliminate this bias, only the most representative trials of allergic rhinitis were included in this study after strict evaluation. Although the number of cases was considerably reduced, the result of ARM is highly reliable, since trials enrolled in this study firmly focus on TCM patterns of allergic rhinitis. Second, the definition of TCM patterns is not exactly the same among these studies, and the basis of pattern differentiation includes Chinese expert consensus on allergic rhinitis in 1997 and 2004, and a textbook of TCM otolaryngology. This disadvantage was largely overcome by examining the descriptions of patterns in every trial and validating them by TCM doctors. Furthermore, results of statistical analysis on large-scale pooled clinical trials are similar to the consensus among TCM experts and thus can overcome the variability of patterns differentiation. 

## 5. Conclusion

Core TCM patterns were explored in this study by applying ARM to clinical trials of allergic rhinitis, and the summarized result is comparable to hospital-based data. A younger patient population and greater severity of nasal stuffiness were associated with the most important patterns, lung or spleen with qi deficiency. Future investigations of TCM treatment for allergic rhinitis can be designed on the basis of these results, and may help define a specific TCM pattern.

## Figures and Tables

**Table 1:** All TCM patterns of 23 clinical studies for allergic rhinitis.

Patterns	Number of patients	Percentage
Lung qi deficiency	620	23.95%
Spleen qi deficiency	589	22.75%
Lung yang deficiency pattern with wind-cold assailing the lung	382	14.75%
Phlegm-heat obstructing the lung	232	8.96%
Dampness-phlegm obstructing the lung	210	8.11%
Kidney yang deficiency	185	7.15%
Kidney qi deficiency	104	4.02%
Dual deficiency of the lung-spleen qi	57	2.20%
Dual deficiency of the spleen-kidney qi	54	2.09%
Qi stagnation and blood stasis	46	1.78%
Dual deficiency of the lung-kidney qi	42	1.62%
Lung-kidney yin deficiency	27	1.04%
Lung-spleen yang deficiency	19	0.73%
Dual deficiency of qi and yin of lung	17	0.66%
Blood stasis	5	0.19%

Total	2589	

**Table 2:** The 10 most common relationships of TCM patterns among 23 clinical studies for allergic rhinitis.

Relationship of pattern	Support	Confidence
Lung with qi deficiency	29.50%	47.35%
Spleen with qi deficiency	28.98%	97.63%
Lung with cold	15.40%	24.72%
Heat with lung	8.61%	100.00%
Phlegm-dampness with lung	7.79%	100.00%
Lung with qi deficiency	7.64%	48.70%
Kidney with cold	7.05%	44.92%
Lung and spleen with qi deficiency	4.71%	86.99%
Kidney and spleen with qi deficiency	2.00%	100.00%
Kidney and lung with qi deficiency	1.78%	64.00%

**Table 3:** Characteristics of TCM patterns of 205 allergic rhinitis patients at Chang Gung Memorial Hospital.

	Lung qi deficiency	Dual deficiency of the lung-spleen qi	Kidney qi deficiency	*P* value
Number of cases	137	55	13	
Age, mean ± SD^†^	29.07 ± 13.17	25.29 ± 12.03	57.53 ± 12.96^∗^	<0.0001
Patients gender				0.690
Male	58	27	6	
Female	79	28	7	
Parents' history of allergic diseases				0.234
None	70	24	9	
One	58	24	2	
Both	9	7	2	
Personal history				
Asthma	16	6	2	0.903
Atopic dermatitis	21	12	1	0.370
Urbanization level				0.423
Urban	93	32	9	
Rural area	44	23	4	
IgE level (IU/mL) mean ± SD^†^	335.05 ± 456.07	420.90 ± 778.82	255.744 ± 433.59	0.494
MAST allergy test (positive/negative)	60/19	25/12	2/4	0.070
Symptom severity, mean ± SD^†^	4.33 ± 2.08	4.64 ± 2.05	3.15 ± 2.23	0.072

*P value < 0.0001 compared to another two groups.

^†^ SD: standard deviation.

**Table 4:** Relations between severity of symptoms and TCM patterns.

	Lung qi deficiency or spleen qi deficiency^‡^	Kidney qi deficiency	*P* value
	*N* = 192	*N* = 13	
Total score, mean ± SD^†^	4.42 ± 2.07	3.15 ± 2.23	0.068
Sneezing, mean ± SD^†^	1.39 ± 0.98	1.08 ± 1.19	0.278
Running nose, mean ± SD^†^	1.68 ± 1.02	1.46 ± 1.05	0.453
Stuffiness, mean ± SD^†^	1.35 ± 0.89	0.62 ± 0.65	0.004^∗^

Key: *P value < 0.05; ^†^SD: standard deviation; ^‡^combination of “lung qi deficiency” and “dual deficiency of the lung-spleen qi” groups.

**Table 5:** Clinical trials of allergic rhinitis included in this study^∗^.

Study	Number of patients	Age (yr; range)	Patterns description (cases)
Yang et al. [35]	216	36.2; 7–63	Lung qi deficiency: 100
			Spleen qi deficiency: 71
			Kidney qi deficiency: 45

Liu et al. [36]	242	42.6^†^	Lung yang deficiency pattern with wind-cold assailing the lung: 167
			Phlegm-heat obstructing the lung: 23
			Spleen qi deficiency: 32
			Kidney yang deficiency: 20

Tang et al. [37]	70	28.95^†^	Lung yang deficiency pattern with wind-cold assailing the lung: 24
			Phlegm-heat obstructing the lung: 16
			Spleen qi deficiency: 20
			Kidney yang deficiency: 14

Qiu et al. [38]	256	32.52; 7–70	Lung qi deficiency: 124
			Phlegm-heat obstructing the lung: 32
			Spleen qi deficiency: 72
			Kidney yang deficiency: 28

Lu et al. [39]	106	31, 4–82	Lung qi deficiency: 60
			Dual deficiency of the lung-spleen qi: 21
			Dual deficiency of the lung-kidney qi: 25

^∗^ Studies without titles or an abstract in English are not listed in this table.

^†^ Range of age was not provided by the authors.

**Table 6:** Symptom severity assessment of allergic rhinitis.

Symptoms	Presentation	Score
Sneezing	None	0
	1–5 times/day	1
	6–10 times/day	2
	>10 times/day	3
Runny nose	None	0
	1–5 times/day	1
	6–10 times/day	2
	>10 times/day	3
Stuffiness	None	0
	Mild, without mouth-breathing	1
	Moderate, with occasional mouth-breathing	2
	Severe, with frequent mouth-breathing	3

## Appendix

For more details see Tables 5 and 6.
